# Threonine aldolases: perspectives in engineering and screening the enzymes with enhanced substrate and stereo specificities

**DOI:** 10.1007/s00253-015-7218-5

**Published:** 2016-01-26

**Authors:** Kateryna Fesko

**Affiliations:** Institute of Organic Chemistry, Graz University of Technology, Stremayrgasse 9, A-8010 Graz, Austria

**Keywords:** Threonine aldolase, Aldol reactions, Screening, Enzyme engineering, Biocatalysis

## Abstract

Threonine aldolases have emerged as a powerful tool for asymmetric carbon-carbon bond formation. These enzymes catalyse the unnatural aldol condensation of different aldehydes and glycine to produce highly valuable β-hydroxy-α-amino acids with complete stereocontrol at the α-carbon and moderate specificity at the β-carbon. A range of microbial threonine aldolases has been recently recombinantly produced by several groups and their biochemical properties were characterized. Numerous studies have been conducted to improve the reaction protocols to enable higher conversions and investigate the substrate scope of enzymes. However, the application of threonine aldolases in organic synthesis is still limited due to often moderate yields and low diastereoselectivities obtained in the aldol reaction. This review briefly summarizes the screening techniques recently applied to discover novel threonine aldolases as well as enzyme engineering and mutagenesis studies which were accomplished to improve the catalytic activity and substrate specificity. Additionally, the results from new investigations on threonine aldolases including crystal structure determinations and structural-functional characterization are reviewed.

## Introduction

**Scheme 1:**
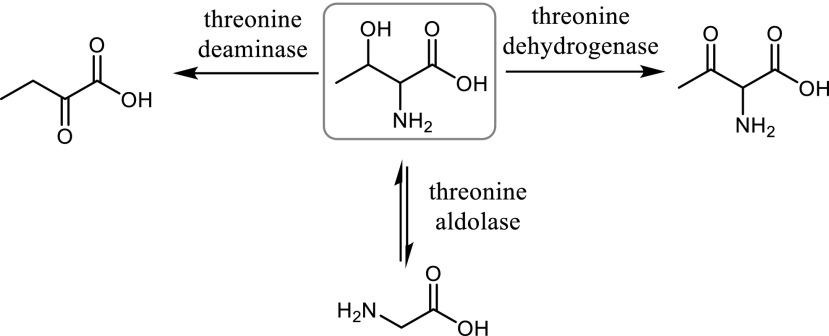
Three main pathways for threonine degradation

Threonine serves as the sole source of carbon and nitrogen for the growth of a wide variety of organisms. Several enzymes are involved in the threonine metabolism. Some enzymes participate in the biosynthesis of threonine from aspartic acid via homoserine, whereas others are responsible for the degradation of threonine (Scheme 1). The retro-aldol cleavage of threonine to form glycine and acetaldehyde is catalysed by threonine aldolases (TAs). TA is also one of several enzymes participating in the alternative pyridoxal-5′-phosphate synthesis pathway, where it catalyses the condensation of glycolaldehyde with glycine (Kim et al. 2010).

TAs are highly selective at the α-carbon of threonine, and thus, l- and d-specific TAs have been recognized. The different selectivities at the β-carbon of threonine for lTA result in either l-, l-*allo*- or l*low-specificity* TA. The last type does not show any selectivity. Only d low-specificity TAs have been found in nature so far. Besides its natural substrate threonine, TAs accept a wide range of β-hydroxy-α-amino acids but generally possess moderate selectivity at *C*_β_ for non-natural substrates. The genes encoding lTA have been found and isolated from bacteria, plants and yeast (Liu et al. 2000). In contrast to lTA, only a few native dTA genes have been reported so far. TAs use pyridoxal-5′-phosphate (PLP) as a cofactor for its activity. Additional to the PLP cofactor, divalent metal ions such as Co^2+^, Ni^2+^, Mn^2+^ or Mg^2+^ are needed to maintain the catalytic activity of dTA (Liu et al. 1998). Besides TAs, another PLP-dependent enzyme, the serine hydroxymethyltransferase (SHMT), catalyses the retro-aldol cleavage of l-β-hydroxy-α-amino acids similar to lTAs in an alternative route (Vidal et al. 2005; Gutierrez et al. 2008).

**Scheme 2:**
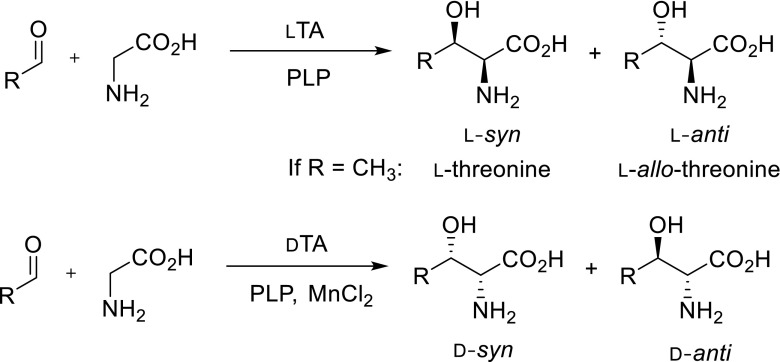
Synthesis of *β*-hydroxy-*α*-amino acids using l-threonine aldolase and d-threonine aldolase

Due to the reversibility of the retro-aldol reaction, the opportunity to use TAs for the biocatalytic production of optically pure non-natural amino acids is of great potential (Scheme 2). For many years, TAs and SHMT have been studied for the asymmetric synthesis of l- and d-β-hydroxy-α-amino acids starting from achiral aldehydes and glycine whereby two chiral centres are formed (Fesko and Gruber 2013). These compounds are components of many complex natural products such as antibiotics and immunosuppressants (e.g. mycestericin D 25, vancomycin, echinocardin D, cyclosporin, katanosin, polyoxin D, empedopeptin). The synthetic reactions as well as substrate range of threonine aldolases from different organisms were recently reviewed by Franz and Stewart (2014). The yields for direct synthesis strongly vary with the aldehyde used and can be improved by using an excess of glycine in order to shift the unfavourable reaction equilibrium to the aldol side. The aldol condensation catalysed by TAs typically leads to a thermodynamically controlled mixture of *syn*/*anti*-isomers of a product. A higher ratio of diastereomers can be usually obtained under conditions of kinetic control, however, with reduced yields (Steinreiber et al. 2007a, b; Fesko et al. 2008).

The currently described protocols for the enzymatic synthesis of β-hydroxy-α-amino acids suffer from a limited substrate range allowing only glycine as the donor, moderate yields and low stereoselectivities at the β-carbon (diastereoselectivity, *d.e.*) of the product. Thus, the application of TAs was mainly focused on the resolution of chemically produced dl-amino acids and on the direct synthesis of threonine or β-phenylserine analogues. In order to overcome the thermodynamic limitations, TAs are often applied in cascade reactions, where the formed amino acid is further converted via additional enzymatic or chemical steps (Baer et al. 2010; Soler et al. 2014; Schmidt et al. 2015). The limitation of rigid donor specificity was recently overcome by isolation of natural TAs with broad donor specificity lTA from *Aeromonas jandaei* and dTA from *Pseudomonas* sp., which accept alanine and serine as donors; thus, l- and d-α-quaternary-α-amino acids were produced (Fesko et al. 2010). In order to improve the stereoselectivity and substrate specificity of TAs, directed evolution techniques as well as search for promiscuous activities could be promising strategies (Wildmann et al. 2012; Davids et al. 2013). This review briefly summarizes the screening and selection methods used in search of new threonine aldolases as well as new structural-functional knowledge which might be helpful for the design of new activities. Despite of some limitations which arise during synthesis, the engineering of this enzyme class was pursued only in a few cases and most of the recent research which lies on the threonine aldolases was focused on finding new wild-type enzymes with improved properties.

## Isolated and described threonine aldolases

Threonine aldolases are widespread in nature and can be found in many organisms. The identification of new TA genes by screening the genomic libraries, isolation from environmental samples and database search for homologous enzymes are currently the most often applied methods. The UniProt database contains more than 6000 genes encoding TAs, which were annotated based on sequence similarities. However, only 1/10 of these genes have been reviewed so far and even less were isolated and tested in biocatalytic reactions. The mining of the sequence database space proved to be an efficient method for the identification and isolation of new threonine aldolase genes. Identified homologous genes are usually typically recombinantly produced in *Escherichia coli* and then their biochemical properties are investigated. A comprehensive review on biochemical properties of the recombinant TAs was published in 2010 (Dückers et al. 2010). In Table 1, we have summarized TAs and SHMT which were isolated, characterized and tested in the synthesis of β-hydroxy-α-amino acids in recent years.

**Table 1:** Threonine aldolases described in literature since year 2010

Enzyme	Organism	NCBI reference sequence	UniProt	PDB	References
L low-specificity TA	*Escherichia coli*	NP_415391	P75823	4LNJ, 4LNM, 4LNL	Baer et al. 2011
					Sagui et al. 2012
					Di Salvo et al. 2014
					Remesh et al. 2015
					Kurjatschij et al. 2014
L-*allo*-TA	*Aeromonas jandaei*	BAA20404	O07051	3WGB	Qin et al. 2014
					Fesko et al. 2010
L low-specificity TA	*Saccharomyces cerevisiae*	NP_010868	P37303		Baer et al. 2011
L low-specificity TA	*Caulobacter crescentus*	NP_421887	Q9A3V8		Giger et al. 2012
L*-allo*-TA	*Thermatoga maritima*	NP_229542	Q9X266	1JG8, 1LW5, 1M6S, 2FM1	Tibhe et al. 2013
					Wieteska et al. 2015
L low-specificity TA	*Ashbya gossypii*	AJ005442	O74267		Barig et al. 2014
l-allo-TA	*Aeromonas veronii*	WP_005351141	K1IGM6		Fesko et al. 2015
lTA	*Cronobacter sakazakii*	WP_004386728	F8YB91		Fesko et al. 2015
lTA	*Shewanella loihica*	WP_011864957	A3QC27		Fesko et al. 2015
l-*allo*-TA	*Geobacter sulfurreducens*	WP_010943783	Q747V3		Fesko et al. 2015
lTA	*Raoultella ornithinolytica*	WP_015584808	A0A0D7EGR3		Fesko et al. 2015
SHMT	*Escherichia coli*	NP_417046	P0A825	1DFO, 3G8M	Zhao et al. 2011
SHMT	*Streptococcus thermophilus*	WP_011227085	Q5M0B4	4WXB	Soler et al. 2014
					Hernandez et al. 2015
dTA	*Pseudomonas aeruginosa*	WP_012077937	A6VED3		Fesko et al. 2015
dTA	*Pseudomonas protegens*	WP_015636124			Fesko et al. 2015
dTA	*Singularimonas variicoloris*	WP_020650309			Fesko et al. 2015
dTA	*Alcaligenes xylosoxidans*			4 V15	Uhl et al. 2015
					Goldberg et al. 2015
dTA	*Pseudomonas* sp*.*	WP_032861317			Fesko et al. 2010
dTA	*Achromobacter xylosoxidans*	BAA86032	Q9RBG6		Soler et al. 2014
dTA	*Arthrobacter* sp*.*	BAA31547	O82872		Goldberg et al. 2015

Fesko et al. have identified a set of new l- and dTAs from genomic databases using previously described threonine aldolases as templates for the BLAST search (Reisinger et al. 2007; Fesko et al. 2008). The overexpression of recombinant TAs usually proceeds at high levels, thus making them promising biocatalysts for industrial application. Further screening of the expressed TAs for substrate promiscuity revealed novel previously not described activities. lTA from *A*. *jandaei* and dTA from *Pseudomonas* sp. were found to accept d-alanine, d-serine and d-cysteine as donors, whereas formerly TAs had been known to be highly specific towards glycine donor (Fesko et al. 2010). Subsequent search for homologues using these enzymes as templates could reveal a set of other lTAs and dTAs with broad donor specificities (Fesko et al. 2015). The respective genes were selected based on 50–80 % sequence similarities to the templates. Most of the selected genes were initially annotated as putative threonine aldolases in case of lTA genes or as putative alanine racemase in case of dTA genes. However, the activities of the identified new TAs are often in a similar range compared to the template enzymes.

In general, TAs from bacteria and yeast have been mostly applied to the biocatalytic investigation, whereas the sequence space from other organisms (e.g. archaea or fungus) has not been exploited yet, which in the future might reveal enzymes with distinct properties (Liu et al. 2015). Liu et al. have performed a systematic phylogenetic analysis of TAs. The results proved that l- and dTAs arise from two phylogenetically unique families. Moreover, lTAs are derived from two distinct families that share low sequence similarity with each other but likely have the same structural fold, suggesting a convergent evolution of these enzymes. The first TA family contains enzymes of both prokaryotic and eukaryotic origins, whereas the second TA family contains only prokaryotic enzymes. The authors concluded that lTAs within the first cluster are more specific towards the β-carbon of threonine and include the l-*allo*-threonine aldolases, whereas the second cluster contains low-specificity threonine aldolases with no selectivity at the β-carbon or preference of l-threonine over l-*allo*-threonine (Liu et al. 2015). Isolation of enzymes from both families and their functional comparison may reveal in future important prerequisites for the stereocontrol in TAs.

## Screening and selection methods

**Fig. 1:**
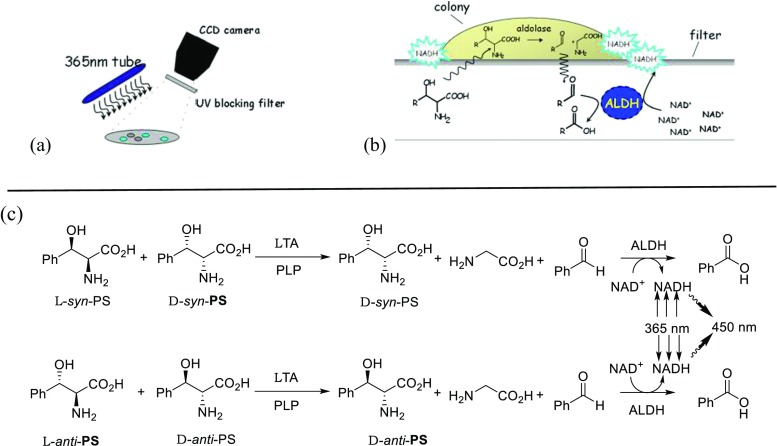
Screening for improved diastereoselectivity by aldol cleavage of *syn*/*anti*-β-phenylserine (*PS*): **a** recording device; **b** colony screening and **c** chemical reaction being involved

One of the main limitations for the application of TAs in the synthesis of β-hydroxy-α-amino acids is their low stereoselectivity at the β-carbon resulting usually in mixture of *syn*/*anti*-isomers. Enzyme specificity can be altered by using directed evolution and rational design techniques; however, this requires a high-throughput screening methodology. The currently described screening approaches of TA variants results generally in a low throughput due to the number of variants that can be screened. In most cases, the screening of the TA variants was done using the HPLC or spectrophotometric methods (Dückers et al. 2010). The latest screening protocol is based on the detection of acetaldehyde formed by retro-aldol cleavage of threonine. In the coupled enzymatic assay, the aldehyde is further converted to ethanol by an alcohol dehydrogenase catalysed reduction of NADH, which is monitored at 340 nm. This method was applied for the high-throughput screening of lTA-producing strains (Liu et al. 2014). If not acetaldehyde, but aromatic aldehydes are formed in the cleavage reaction, this can be directly measured at 279 nm. This method was applied for the identification of variants with improved activity and stability towards l-*syn*-β-3,4-dihydroxyphenylserine (l-*threo*-DOPS) as substrate (Baik et al. 2007). A high-throughput assay that directly interrogates stereoselectivity was designed by Reisinger et al. (2006). The screening system was developed on the colony level (Fig. 1) for the retro-aldol cleavage of *syn*- and *anti*-β-phenylserine. The formation of benzaldehyde was detected by a coupled oxidation of benzaldehyde to benzoic acid (catalysed by aldehyde dehydrogenase, ALDH). In doing so, the formed NADH is recorded through its fluorescence at 450 nm upon excitation at 365 nm. In contrast to other methods, *anti*-β-phenylserine and thereafter *syn*-β-phenylserine are screened on the same plate, and both images are then overlaid. A washing step between two screening substrates is mandatory to remove *anti*-β-phenylserine and NADH. The mutants with improved diastereoselectivities for the cleavage of β-phenylserine have been selected; however, further investigation showed no improvement of selectivity in the synthetic reaction.

The drawback of the described screening methods is that they apply retro-aldol reactions, where the kinetics are different compared to the aldol reaction. Moreover, the ratio of diastereomers is often different in the aldol and retro-aldol reaction. Consequently, the more important thermodynamically driven formation of isomers in the synthesis direction remains unobserved, because during the screening only kinetically controlled reaction is investigated (Fesko et al. 2008; Franz and Stewart 2014). Additionally, the aldol synthesis proceeds usually with large excess of a donor (glycine), which is also not simulated by the screening techniques.

**Fig. 2:**
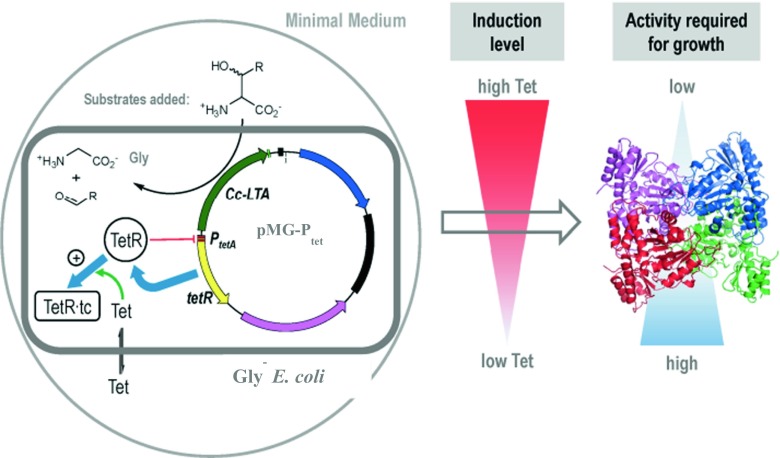
In vivo selection strategy for threonine aldolase activity based on the cleavage of β-hydroxy-α-amino acids. The threonine aldolase gene Cc-lTA is expressed from pMG-Ptet in the glycine auxotroph using the tetracycline-inducible Ptet system, which provides regulation of the stringency of the selection system by varying the inducer concentration (Giger et al. 2012)

In contrast to screening, where establishing a rapid and sensitive screening system to screen more than 10^4^ mutant enzymes is challenging, the selection technology is a useful alternative. The classical in vivo selection approach relies on the growth of auxotrophic cells, where under appropriate conditions only those clones appear that are actually desired, which allows to screen libraries with >10^6^ members (the size of the library is limited preliminarily by the transformation efficiency). Giger et al. have developed a new genetic selection system for TAs where the retro-aldolase activity was directly linked to the cellular growth by simultaneous inactivation of four essential genes involved in glycine biosynthesis in *E. coli*. (Giger et al. 2012). The glycine auxotrophic host strain can grow only on a minimal medium supplemented with glycine or by coupling with a glycine-producing enzyme activity, such as expression of a gene encoding an aldolase that converts β-hydroxy-α-amino acids, provided in the medium, to glycine and the corresponding aldehyde. The aldolase gene was placed under the control of the tetracycline-inducible P_tet_ system to enable the variation of intracellular enzyme concentration and, thus, adjust the stringency of the selection system (Neuenschwander et al. 2007). High tetracycline concentration leads to the expression of the large amounts of an enzyme, so even weak catalysts should be able to complement the glycine deficiency, whereas at low tetracycline concentrations, relatively little enzyme is produced and highly active aldolases are needed to achieve wild-type levels of growth (Fig. 2). The described selection system was applied for the characterization of four catalytic residues, which were simultaneously mutated in the active site of lTA from *Caulobacter crescentus* (see below). In principle, this method allows screening TA genes towards different β-hydroxy-α-amino acid substrates. However, the system is restricted towards only glycine producing reactions.

**Fig. 3:**
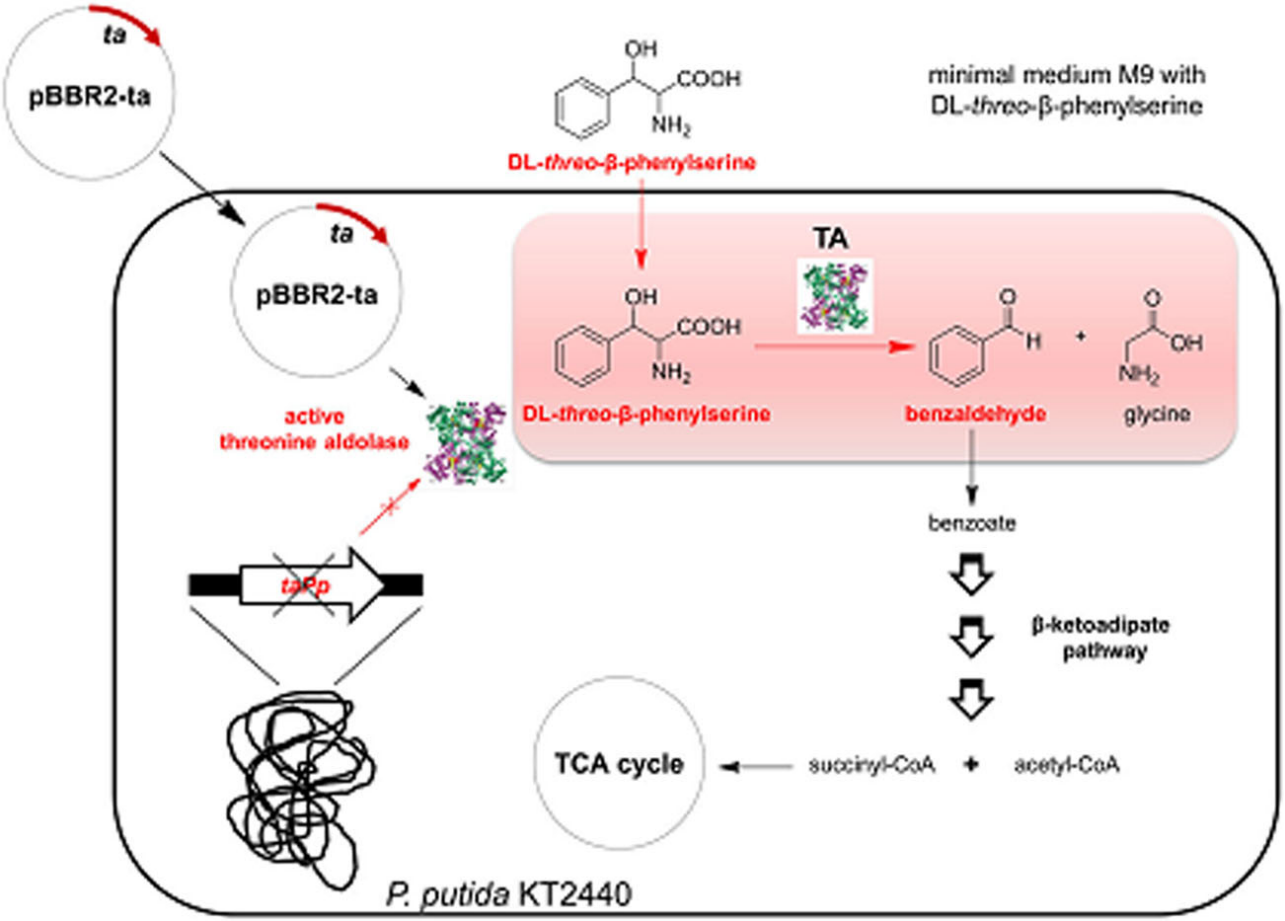
In vivo selection strategy for threonine aldolase activity based the cleavage of β-phenylserine. The threonine aldolase gene TA is expressed from pBBR2-ta in the *Pseudomonas putida* KT2440 strain with deletion of inherent TA gene (*taPp*) (Figure taken with permission from Springer Publisher) (Bulut et al. 2015)

Another growth-dependent selection system in *Pseudomonas putida* KT2440 for the identification of l-threonine aldolases with improved properties (e.g. higher diastereoselectivities or/and activities) was developed by Bulut (Bulut et al. 2015). The strain can use benzaldehyde, the cleavage product of β-phenylserine by TA, as sole carbon source via the β-ketoadipate pathway. By introducing and expressing genes encoding TA to the strain, the growth on minimal media supplemented with dl-β-phenylserine can be monitored. The inherent gene encoding TA in *P*. *putida* was deleted in order to create a highly efficient selection system (Fig. 3). Surprisingly, only the selection strains with a plasmid bearing lTA genes were able to grow, whereas the corresponding strain with dTA gene showed no growth. This was explained by a lack of transport of d-enantiomer of amino acids to the cells to be taken up by TAs. The described selection method in theory can be applied for the screening of α-substituted β-phenylserines to find novel TAs with broad donor specificity.

Lee et al. have developed a growth-dependent selection system based on the consumption of toxic acetaldehyde by *E. coli* cells containing TA genes (Lee et al. 2003). Clones with high TA activity consume acetaldehyde faster, thereby allowing the faster growth of the cells. In contrast to the above described selection systems, this method allows to screen TAs in the synthetic direction. By combining directed evolution and the described selection system, variants of lTA from *Pseudomonas aeruginosa* with a twofold increase of catalytic activity compared to the wild type were selected. However, the applicability of this method with other substrates was not tested.

## Enzyme engineering

Taking into account that TAs are potentially interesting for industrial applications, several attempts were performed to increase their stability. Using rational design, the thermal stability of thermophilic lTA from *Thermatoga maritima* was improved by incorporation of salt and disulphide bridges between subunits (Wieteska et al. 2015). The variant P56C showed enhanced stability at 80 °C by 15 % compared to the wild type, whereas the W86E variant displayed improved activity in the cleavage of l-threonine, but no change in thermostability. In another attempt, the thermostability of lTA from *Streptomyces coelicolor A3* was improved by error-prone PCR. The variants were screened for the ability to cleave L-threonine at 65 °C (Baik et al. 2007). Four variants containing mutations H177Y, A169T, D104N and F18I were selected with improved thermostability compared to the wild-type enzyme.

Different techniques were tested to immobilize the aldolases for the production of β-hydroxy-α-amino acids. The immobilization of lTA from *E*. *coli* on different supports was studied to increase the stability of the enzyme. The entrapment into a matrix of orthosilicate gel was the most promising immobilization method (Kurjatschij et al. 2014). In another study, lTA from *T*. *maritima* was immobilized on Eupergit® oxirane acrylic beads and applied for the synthesis of l-β-phenylserine in a microreactor (Tibhe et al. 2013). Although the product yield was lower compared to the reactions with free enzymes, the stability of the catalyst at 80 °C was improved (Fu et al. 2012). Zhao et al. used immobilized *E. coli* cells containing the SHMT gene for the resolution of dl-β-phenylserine derivatives. The immobilized cells were continuously used 10 times, yielding an average conversion rate of 60.4 % (Zhao et al. 2011).

Only relatively few examples have been reported, where the stereoselectivity and catalytic activity were improved by means of enzyme engineering. Phenylserine derivatives are the main targets for TAs because of their component of several semisynthetic β-lactam antibiotics, as well as they represent the hydroxyl-substituted analogues to l-*threo*-DOPS, a drug used in the treatment of Parkinson’s disease. Thus, the diastereoselectivity of lTA from *S*. *coelicolor* towards l-*threo*-DOPS was enhanced by a directed evolution approach (Gwon and Baik 2010). The best variant with combined mutations Y34C/Y39C/A48T/Y306C showed 55.4 % *d.e.* after 80 h during the batch reactor. Later, this mutant was used for the whole-cell aldol condensation bioreactor to produce l-*threo*-DOPS (Gwon et al. 2012).

SHMT from *Streptococcus thermophilus* was engineered to catalyse the stereoselective synthesis of l-α-methyl- and α-hydroxymethyl-α-amino acids similar to published lTAs with broad donor specificity. The single mutation Y55T which was reported to be involved in the donor specificity increased the donor specificity towards d-serine, whereas only glycine and d-alanine (with less activity) were accepted by the wild-type enzyme (Hernandez et al. 2015).

## Structure, catalytic mechanism and mutagenesis studies

**Fig. 4:**
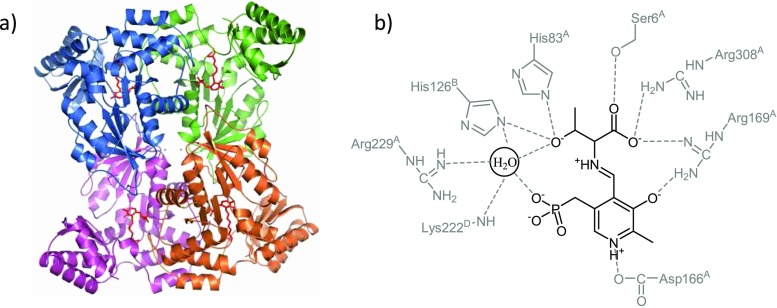
Schematic representation of the structure and active site of LTA from *Escherichia coli* (pdb code 4LNJ): **a** tetrameric structure with internal aldimine PLP-Lys197 (*red*) in each active site and **b** stabilization of substrate-PLP complex

lTA and dTA belong to different fold types of PLP-dependent enzymes and, thus, have distinct structures and evolutional origins. The first crystal structure of lTA was published for the *T*. *maritima* (tmTA) enzyme in 2002 (Kielkopf and Burley 2002). LTAs belong to the aminoaspartate fold family (fold type I) of PLP enzymes. The enzyme functions as a homotetramer, where each monomer consists of a small and a large domain (Fig. 4a). The active site is located at the dimer interface between two subunits. The PLP cofactor is bound via a Schiff base to the catalytic lysine (Lys199 in tmTA). It was postulated that the β-hydroxyl group of the l- and l-*allo*-threonine substrate interacts with the active-site His83 and His125 residues and an ordered water molecule, whereas the side chain of His83 might act as a general acid-base group in the catalytic mechanism and may influence the stereoselectivity at the β-carbon of threonine (Kielkopf and Burley 2002). Di Salvo et al. have recently investigated the impact of two histidines on the catalytic activity and stereoselectivity in the lTA from *E*. *coli* (ecTA) (di Salvo et al. 2014). Mutational studies on His83 and His126 (corresponds to His125 in tmTA) alone, or a double His83/His126 mutation, were performed, and neither of these mutations was lethal for the enzyme activity. Substitution of His83 to Asn or Phe had a detrimental effect mainly due to the release of PLP from the active site and thus precipitation of an apo-enzyme unless an excess of PLP was added. The structural studies of the wild-type enzyme ecTA co-crystallized with l-serine and l-threonine in combination with active-site mutants clearly showed that neither of these residues is the catalytic base. On the other hand, the authors have assumed that the conserved active-site water molecule may act as the catalytic base in the retro-aldol cleavage, where His83 and His126 participate in water coordination to enhance its basicity (Fig. 4b). This was also confirmed by mutation of another relevant conserved active-site residue in TAs—Lys222, which is involved in the interaction network to keep the base water molecule in place. This mutation to alanine resulted in 10 times reduced activity. Although neither His83 nor His126 act as a catalytic base, both residues clearly play some role in the substrate binding and the determination of the stereospecificity of the enzyme. The mutations H83F and H126F increased the specificity towards L-threonine, whereas H83N and H126N mutants did not influence the stereo preferences. Further, mutations at Phe87 were investigated to affect the diastereoselelectivity; however, the study did not reveal a clear effect (di Salvo et al. 2014). Thus, the design of diastereoselectivity of lTAs is still a challenging task, for which a deep understanding of the catalytic mechanism is required. The substrate recognition and reaction specificity seem to be guided by the overall microenvironment that surrounds the substrate at the enzyme active site, rather than by one or more specific residues.

Another crystal structure of ecTA at low pH with PLP as an internal aldimine and uncleaved l-serine as substrate was published recently (Remesh et al. 2015). The authors have compared the structure with those obtained at physiological pH and investigated the molecular basis for the inactivation of ecTA at low pH. At pH 5.6, the substrate amino acid is protonated thereby causing a non-productive orientation, where the α-amino group and carboxylate group are in wrong positions. In this case, the carboxy group of the substrate is oriented and stabilized by the His residues, rather than by Arg169 and Arg308 residues at neutral pH (Fig. 4b). Moreover, the active-site residues His83 and His126 are protonated at low pH preventing the characteristic nucleophilic attack of the α-amino group of substrate to form the external aldimine with PLP. Thus, low pH changes the charge distribution in the active site and results in non-covalent binding of a substrate.

**Fig. 5:**
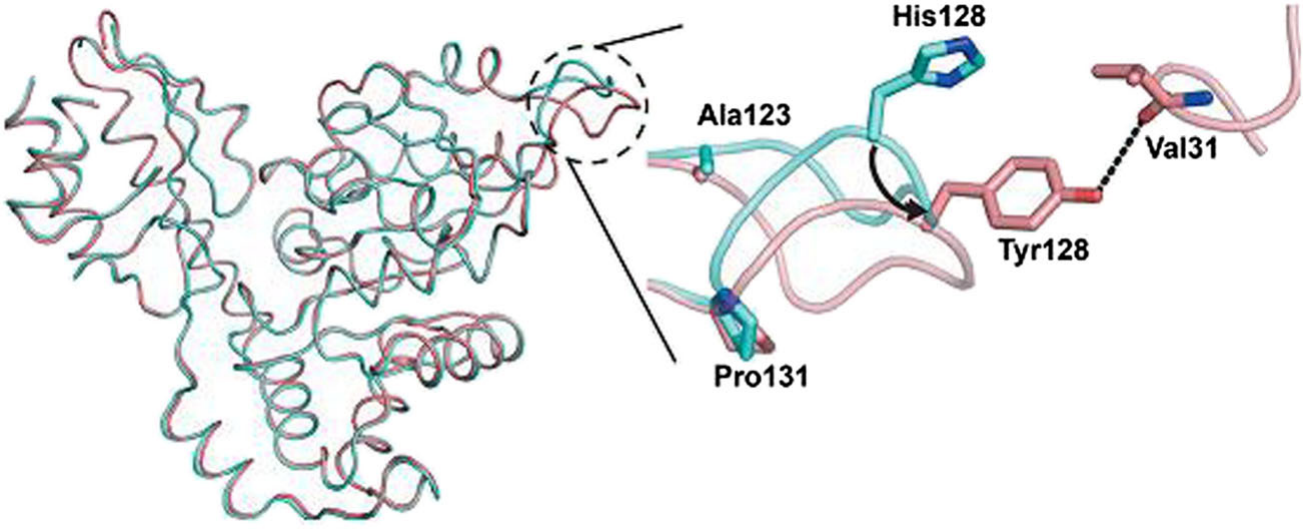
Superposition of the structures of L-*allo-*TA *Aeromonas jandaei* wild type (*cyan*; pdb code 3WGB) and H128Y/S291R double mutant (*salmon*; pdb code 3WGC). The different conformation is indicated by a *dashed circle* (Figure taken with permission from Tanokura) (Qin et al. 2014)

Another investigation on the role of His128 was done for l-*allo*-TA *A*. *jandaei* ajTA (corresponding to His125 in tmTA and His126 in ecTA) by Tanokura (Qin et al. 2014). In order to find the residues which are important for the catalytic activity, a mutant library was created by random mutagenesis and 3000 clones were screened for increased activity towards the cleavage of l-*allo*-threonine and l-threonine. Only two mutations were found in the best variants: H128Y and S292R. The authors postulated that the His128 residue regulates the substrate specificity and might influence the stereospecificity. Indeed, the mutant H128Y showed the highest activity towards the two substrates, with an 8.4-fold increase towards the unfavoured isomer l-threonine and a 2.0-fold increase towards l-*allo*-threonine compared to the wild-type enzyme (Table 2). The double mutant H128Y/S292R was crystallized to elucidate the structural basis of its stereo- and substrate specificity. The influence of the mutations on the substrate specificity was explained by structural changes of the enzyme. Thus, His128 moves 4.2 Å outwards from the active site by mutation on Tyr, thereby changing the substrate stereoselectivity (Fig. 5). The influence of S292R was not clear as the crystal structure was disordered at this region. Further, the saturation mutagenesis at His128 revealed that most substitutions by hydrophobic residues improve the activity towards l-threonine presumably due to a hydrophobic interaction between the methyl group of L-threonine and the side chains of the mutated residues. In general, the substrate specificity in ajTA is influenced by several residues which directly interact with a substrate, e.g. His85, Tyr89, His128 as well as other residues, which participate in the hydrogen-bonding network of the active site, e.g. Glu90 and Asp126. Thus, the substrate specificity results from the defined orientation in the active site which is determined by the overall hydrogen network and electrostatic interaction.

**Table 2:** Kinetic parameters and description of mutants of L-threonine aldolases analysed since 2010

Enzyme	Mutation	Substrate	Description			Reference
			kcat, min^−1^	Km, mM	kcat/Km, mM^−1^ min^−1^	
lTA *Escherichia coli*	WT	L-*allo*-Thr	213	0.24	887	Di Salvo et al. 2014
		L-Thr	112	19.4	5.8	
	H126N	L-*allo*-Thr	469	0.96	488	
		L-Thr	262	61	4	
	H126F	L-*allo*-Thr	541	0.2	2705	
		L-Thr	360	1.7	212	
	H83N	L-*allo*-Thr	77	1.7	45	
		L-Thr	17	38	0.4	
	H83F	L-*allo*-Thr	3.7	7	0.53	
		L-Thr	1.2	7	0.17	
	F87A	L-*allo*-Thr	160	0.31	516	
		L-Thr	43	8.7	4.9	
	F87D	L-*allo*-Thr	117	0.41	285	
		L-Thr	19.8	24.9	0.79	
	H83F/H126F	L-*allo*-Thr	1.7	7.4	0.23	
		L-Thr	0.44	21	0.02	
	K222A	L-*allo*-Thr	80	1.2	67	
		L-Thr	43	72	0.6	
lTA *Aeromonas jandaei*	WT	L-*allo*-Thr	828	0.5	1656	Qin et al. 2014
		L-Thr	38	31.6	1.2	
	H128Y	L-*allo*-Thr	990	0.4	2475	
		L-Thr	108	4.5	24	
	H128Y/S292R	L-*allo*-Thr	1320	0.4	3300	
		L-Thr	128	3.2	40	
	E90A	L-*allo*-Thr			110	
		L-Thr			0.04	
	D126A	L-*allo*-Thr			760	
		L-Thr			0.7	
lTA *Caulobacter crescentus*	WT	L-*allo*-Thr	1800	0.69	2600	Giger et al. 2012
		L-Thr	198	13.6	15	
		L-*threo*-PS	4020	0.035	1*10^5^	
	D176E	L-*allo*-Thr			<0.05	
		L-Thr			<0.05	
		L-*threo*-PS	6	0.24	25	
	D95W	L-*allo-*Thr	1140	2.5	456	
		L-Thr	576	44	13	
		L-*threo*-PS	1020	0.78	1300	
	D95L	L-*allo*-Thr	1320	12	110	
		L-Thr	228	53	4.3	
		L-*threo*-PS	1380	0.19	7260	
	D95Y	L-*allo*-Thr	1200	2.7	440	
		L-Thr	210	63	3.3	
		L-*threo*-PS	840	0.31	2700	
	D95Y/E96T	L-*allo*-Thr	180	4.4	41	
		L-Thr			0.1	
		L-*threo*-PS	168	0.027	6200	
lTA *Streptomyces coelicolor*	Y39C/Y306C	L-*threo*-DOPS	Fivefold increased diastereoselectivity			Gwon and Baik 2010
lTA *Thermatoga maritima*	W86E	L-Thr	1.5-fold increase in catalytic activity			Wieteska et al. 2015
	T59D	L-Thr	Significant loss of activity			
	V29D; P56C	L-Thr	Increased in stability at 80 °C			
SHMT *Streptococcus thermophilus*	Y55T	L-α-methyl-β-hydroxy-α-amino acids	Increased donor specificity; control of catalytic promiscuity			Hernandez et al. 2015

To a similar conclusion came the authors, trying to identify the residues responsible for the broader donor specificity of ajTA compared to those which only accept glycine as the donor (Fesko et al. 2015). The sequence and crystal structure alignment of available lTAs reveal that the catalytically important residues were conserved among lTAs from different organisms, whereas their orientation in the active site was different in some cases. The conformational changes were found in the region Met281-Arg313, which might be responsible for the substrate recognition as well as for the flexibility of the active site. Moreover, the spatial orientation of Arg313, which participates in the stabilization of the carboxylate group of a donor, was slightly different in all structures. The authors have postulated that these differences may interfere with the hydrogen-bonding network which is necessary for the stabilization of a donor substrate in a catalytically active orientation and the transition state during the enzymatic reaction. However, no clear conclusion on how the donor specificity may be regulated or improved could be made. It becomes obvious that the substrate specificity of TAs is a result of complex interactions in the active site; thus, further investigations on how subtle structural changes in the active site influence the catalytic activity and selectivity are essential for further engineering of the enzyme.

In order to reveal the catalytically important residues in the active site of lTAs, Giger et al. have analysed a multiple sequence alignment of 95 unique l-threonine aldolases available in the UniProt database in conjunction with available structural data and highlighted several highly conserved residues which presumably represent the catalytic core of this enzyme family (Giger et al. 2012). Besides critical lysine which forms the internal aldimine with the PLP (Lys 207 for the lTA from *C*. *crescentus*, ccTA), an aspartate (Asp176) involved in binding the protonated cofactor as well as glycine (Gly177) immediately downstream of the aspartate, a histidine (His91) that π-stacks against the *re* face of the PLP, and the arginine (Arg179) that forms a salt bridge with the substrate carboxylate in the external aldimine intermediate were identified. Furthermore, a mutant library of ccLTA at four target sites (Asp176, His91, Glu96, Asp95) was created by error-prone PCR, and the genes were transferred to the glycine auxotroph *E*. *coli* strain to investigate the influence of the mutations on the activity of LTA which is linked to the growth ability of the cells on minimal media (see above). Interestingly, only His91 (corresponding to His83 in tmTA and ecTA) was absolutely required for the aldolase activity. In contrast, conserved Asp176 can be replaced by glutamate, however, with >5000-fold decrease in efficiency (Table 2). Both, Glu96 and Asp95 were not essential for the catalytic activity; however, they participate in substrate binding and stabilization.

In Table 2, we have summarized the analysis of enzyme activities of TA mutants described in the recent years.

**Fig. 6:**
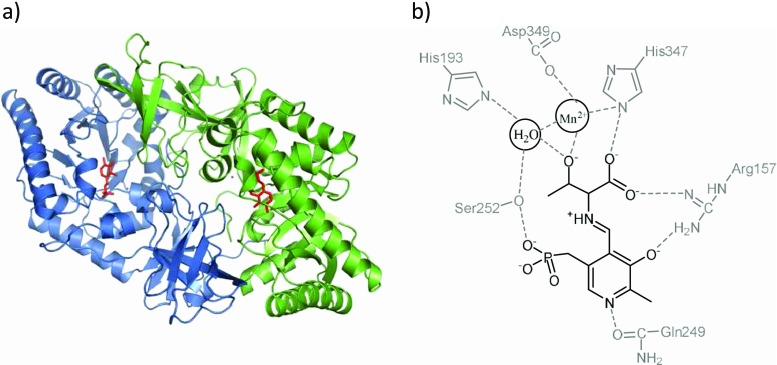
Schematic representation of the structure and active site of DTA from *Alcaligenes xylosoxidans* (pdb code 4V15): **a** dimeric structure with internal aldimine PLP-Lys59 (*red*) in each active site and **b** stabilization of substrate-PLP complex

**Scheme 3:**
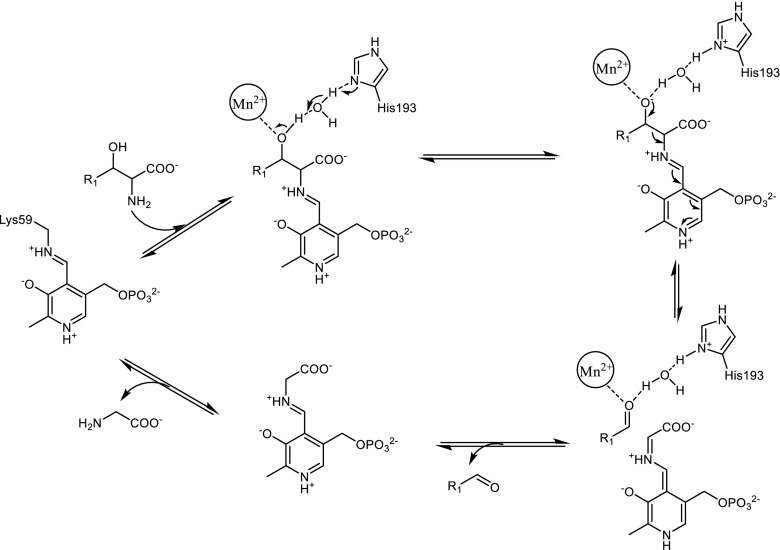
The proposed mechanism of retro-aldol cleavage of β-hydroxy-α-amino acids catalysed by DTA from *Alcaligenes xylosoxidans*

The first crystal structure of d-specific TAs was published recently by Gruber et al. for the dTA from *Alcaligenes xylosoxidans* (axDTA) (Uhl et al. 2015). DTA belongs to the alanine racemase family of PLP-dependent enzymes (fold type III), and its structure is different from l-specific TAs. Nevertheless, the crucial active-site residues of axDTA relative to the PLP cofactor are in approximate mirror symmetry to lTAs. The axDTA was obtained as a stable dimer, which is similar to the dimer of known bacterial alanine racemases and involves non-covalent contacts between both subdomains (Fig. 6a). Moreover, the metal binding site close to the PLP cofactor in the active centre was identified, which is consistent with previous observations that divalent cations are essential for dTA activity. The authors have postulated that the β-hydroxy group of a substrate binds to the metal ion and, likely, activates this group and facilitates its deprotonation by His193 (Fig. 6b). By docking d-*syn* and d-*anti*-β-phenylserine as substrates to the active site of axDTA, the potentially crucial residues for the catalytic activity and substrate binding were identified and a mechanism of catalysis for the degradation of β-hydroxy amino acids catalysed by axDTA was proposed (Scheme 3). The putative mechanism involves a manganese ion as a crucial Lewis acid, which coordinates the β-OH group of the substrate, and His193 as the base. Proton abstraction is mediated more likely by a water molecule similar to lTAs.

## Summary and outlook

Threonine aldolases have been established as efficient biocatalysts for stereoselective carbon-carbon bond formation. The synthetic methods with threonine aldolases are a good alternative to existing chemical methods, as highly functional compounds can be produced under mild conditions without protection/deprotection steps. Due to the existence of enantio-complementary enzymes, the asymmetric synthesis of both l- and d-β-hydroxy-α-amino acids is possible. Moreover, the recent discoveries of TAs and SHMT with broad donor specificities opened the way towards enantioselective syntheses of α-quaternary α-amino acids. The current obstacles in TA application are their limited substrate scope, poor selectivity at the β-carbon of products and unfavourable thermodynamic equilibria leading to low concentrations of products. If the latest can be changed by in situ removal of the product, e.g. by combination of TA-catalysed reactions with additional (irreversible) step, the substrate and stereospecificity can be manipulated by protein engineering techniques. In recent years, scientists have focused on understanding the mechanism of catalysis and function-structure relationships in TAs. The appearance of new crystal structures of lTAs and the first structure of dTA along with mutagenesis studies give a good starting point for the engineering of the catalytic properties of TAs. However, high-throughput screening methodologies in the synthesis directions and/or more general selection methods are still required. On the other hand, exploring the sequence space that has so far not been examined can be applied to screen for novel TAs with desired activities. More detailed investigation of the evolutionary relationship of this enzyme family with other PLP enzymes might be useful to determine the subfamily-specific positions, which are responsible for the reaction and substrate specificity. Moreover, exploring the promiscuity of PLP enzymes may be a promising alternative to find/create TA with novel properties.
